# Perioperative versus adjuvant S-1 plus oxaliplatin chemotherapy for stage II/III resectable gastric cancer (RESONANCE): a randomized, open-label, phase 3 trial

**DOI:** 10.1186/s13045-024-01536-7

**Published:** 2024-04-08

**Authors:** Xinxin Wang, Canrong Lu, Bo Wei, Shuo Li, Ziyu Li, Yingwei Xue, Yingjiang Ye, Zhongtao Zhang, Yihong Sun, Han Liang, Kai Li, Linghua Zhu, Zhichao Zheng, Yanbing Zhou, Yulong He, Fei Li, Xin Wang, Pin Liang, Hua Huang, Guoli Li, Xian Shen, Jiafu Ji, Yun Tang, Zekuan Xu, Lin Chen, on behalf of RESONANCE study group

**Affiliations:** 1 Department of General Surgery, Chinese PLA General Hospital, No. 28 Fuxing Road, Haidian District, Beijing, 100853 China; 2 Department of Gastrointestinal Surgery, Peking University Cancer Hospital, No. 52 Fucheng Road, Haidian District, Beijing, 100142 China; 3 Department of Gastroenterological Surgery, Harbin Medical University Cancer Hospital, No. 150 Haping Road, Nangang District, Harbin, Heilongjiang Province 150081 China; 4 Department of Gastroenterological Surgery, Peking University People’s Hospital, No.11 Xizhimen South Street, Xicheng District, Beijing, 100044 China; 5 Department of General Surgery, Beijing Friendship Hospital, Capital Medical University, No.95 Yongan Road, Xicheng District, Beijing, 100050 China; 6 Department of General Surgery, Zhongshan Hospital, Fudan University, No. 180 Fenglin Road, Xuhui District, Shanghai, 200032 China; 7 Department of Gastric Cancer Surgery, Tianjin Medical University Cancer Hospital, West Huan-Hu Road, Ti Yuan Bei, Hexi District, Tianjin, 300060 China; 8 Department of Surgical Oncology, The First Hospital of China Medical University, No.155 Nanjing Street North, Heping District, Shenyang, Liaoning Province 110002 China; 9 Department of General Surgery, Sir Run Run Shaw Hospital, School of Medicine, Zhejiang University, No.3 East Qingchun Road, Shangcheng District, Hangzhou, Zhejiang Province 310016 China; 10 Department of Gastric Surgery, Liaoning Cancer Hospital and Institute, No.44 Xiaoheyan Road, Dadong District, Shenyang, Liaoning Province 110042 China; 11 Department of General Surgery, The Affiliated Hospital of Qingdao University, No.16 Jiangsu Road, Shinan District, Qingdao, Shandong Province 266000 China; 12 Department of Gastrointestinal Surgery, The First Affiliated Hospital, Sun Yat-sen University, No.58 Zhongshaner Road, Guangzhou, Guangdong Province 510080 China; 13 Department of General Surgery, Xuanwu Hospital, Capital Medical University, No.45 Changchun Street, Xicheng District, Beijing, 100053 China; 14 Department of General Surgery, Peking University First Hospital, No.8 Xishiku Street, Xicheng District, Beijing, 100034 China; 15 Department of Gastrointestinal Surgery, The First Affiliated Hospital of Dalian Medical University, No.222 Zhongshan Road, Xigang District, Dalian, Liaoning Province 116011 China; 16 Department of Gastric Surgery, Fudan University Shanghai Cancer Center, No. 270 Dongan Road, Xuhui District, Shanghai, 200032 China; 17 Institute of General Surgery, General Hospital of Eastern Theater Command of Chinese PLA, No.305 East Zhongshan Road, Xuanwu District, Nanjing, Jiangsu Province 210002 China; 18 Division of Gastrointestinal Surgery, The Second Affiliated Hospital of Wenzhou Medical University, No.109 West Xueyuan Road, Wenzhou, Zhejiang Province 325027 China; 19 Department of General Surgery, Jiangsu Province Hospital, No.300 Guangzhou Road, Gulou District, Nanjing, Jiangsu Province 210029 China; 20 Department of Gastrointestinal Surgery, Peking University International Hospital, No.1 Life Garden Road, Zhongguancun Life Science Park, Changping District, Beijing, 102206 China

**Keywords:** Gastric cancer, Perioperative, Adjuvant, Chemotherapy, S-1, Oxaliplatin

## Abstract

Evidence from Europe shows that perioperative chemotherapy may be beneficial for the treatment of locally advanced gastric cancer, but reliable and robust data is lacking. To rectify this, the phase 3 RESONANCE trial investigated the efficacy and safety of S-1 plus oxaliplatin (SOX) as a perioperative chemotherapy regimen for gastric cancer. This randomized, open-label trial enrolled patients from 19 medical centers with stage II/III resectable gastric cancer who were centrally randomly assigned to either perioperative chemotherapy (PC) arm or adjuvant chemotherapy (AC) arm. Patients in the PC arm received two to four cycles of SOX followed by surgery and four to six cycles of SOX. Patients in the AC arm received upfront surgery and eight cycles of SOX. 386 patients in each group were enrolled and 756 (382 in PC and 374 in AC) were included in the mITT population. The three-year DFS rate was 61.7% in the PC arm and 53.8% in the AC arm (log-rank *p* = 0.019). The R0 resection rate in the PC arm was significantly higher than that in the AC arm (94.9% vs. 83.7%, *p* < 0.0001). There was no difference between two arms in surgical outcomes or postoperative complications. Safety-related data were like the known safety profile. In conclusion, from a clinical perspective, this trial indicated a trend towards higher three-year disease-free survival rate with perioperative SOX in stage II/III resectable gastric cancer with well-tolerated toxicity compared to adjuvant SOX, which might provide a theoretical basis for applying perioperative SOX in advanced gastric cancer patients. (ClinicalTrials.gov NCT01583361)

**Supplementary Information:**

The online version contains supplementary material available at 10.1186/s13045-024-01536-7.

**To the Editor**.

Curative resection is the mainstay for resectable gastric cancer [1]. To further improve survival, multidisciplinary strategies such as perioperative chemotherapy and postoperative chemotherapy have been assessed. The MAGIC study, FNCLCC/FFCD 9703 study, and FLOT4 study have established the rationale for perioperative chemotherapy in western countries, showing better overall survival in perioperative settings than surgery only [2–4]. In contrast, the ACTS-GC trial and CLASSIC trial have solidified adjuvant chemotherapy as a standard treatment in East Asia [5, 6]. Despite these advances, current evidence does not suggest a preferred therapeutic strategy or an optimal chemotherapy regimen. Several studies have shown that the S-1 plus oxaliplatin chemotherapy (SOX) was efficient and well tolerated [7–10]. However, there remains a scarcity of direct comparisons between perioperative and adjuvant chemotherapy using SOX. Therefore, the randomized RESONANCE trial was conducted to compare perioperative with adjuvant SOX chemotherapy in patients with locally advanced gastric cancer. Study Methods were contained in Additional file 1.

Between Sep 1, 2012, and Jul 1, 2019, 772 patients from 19 medical centers were enrolled and randomly assigned to perioperative chemotherapy (PC) arm or adjuvant chemotherapy (AC) arm (Additional file 2: Fig. S1, Table S1). 382 in PC arm receiving preoperative chemotherapy and 374 in AC arm receiving surgical resection formed the modified intention-to-treat (mITT) population (Additional file 2: Table S2). The three-year disease-free survival (DFS) rate was 61.7% (95%CI 56.8-66.6%) in PC group and 53.8% (95%CI 48.8-58.9%) in AC group. The hazard ratio (HR) was 0.76 (95%CI 0.61–0.96) and log-rank *p* = 0.019 (Fig. 1A). Subgroup analysis revealed a significant difference in DFS between the two groups among stage III patients, rather than among stage II patients (Fig. 1B and C, Additional file 2: Fig. S2). In the per-protocol population, which consisted of patients who received surgery and preoperative and postoperative chemotherapy in PC group or postoperative chemotherapy in AC group, the three-year DFS rate was 63.0% (95%CI 58.1-67.9%) in PC group and 55.5% (95%CI 50.3-60.7%) in AC group (HR 0.77, 95%CI 0.61–0.96, *p* = 0.026) (Additional file 2: Fig. S3).

**Fig. 1:**
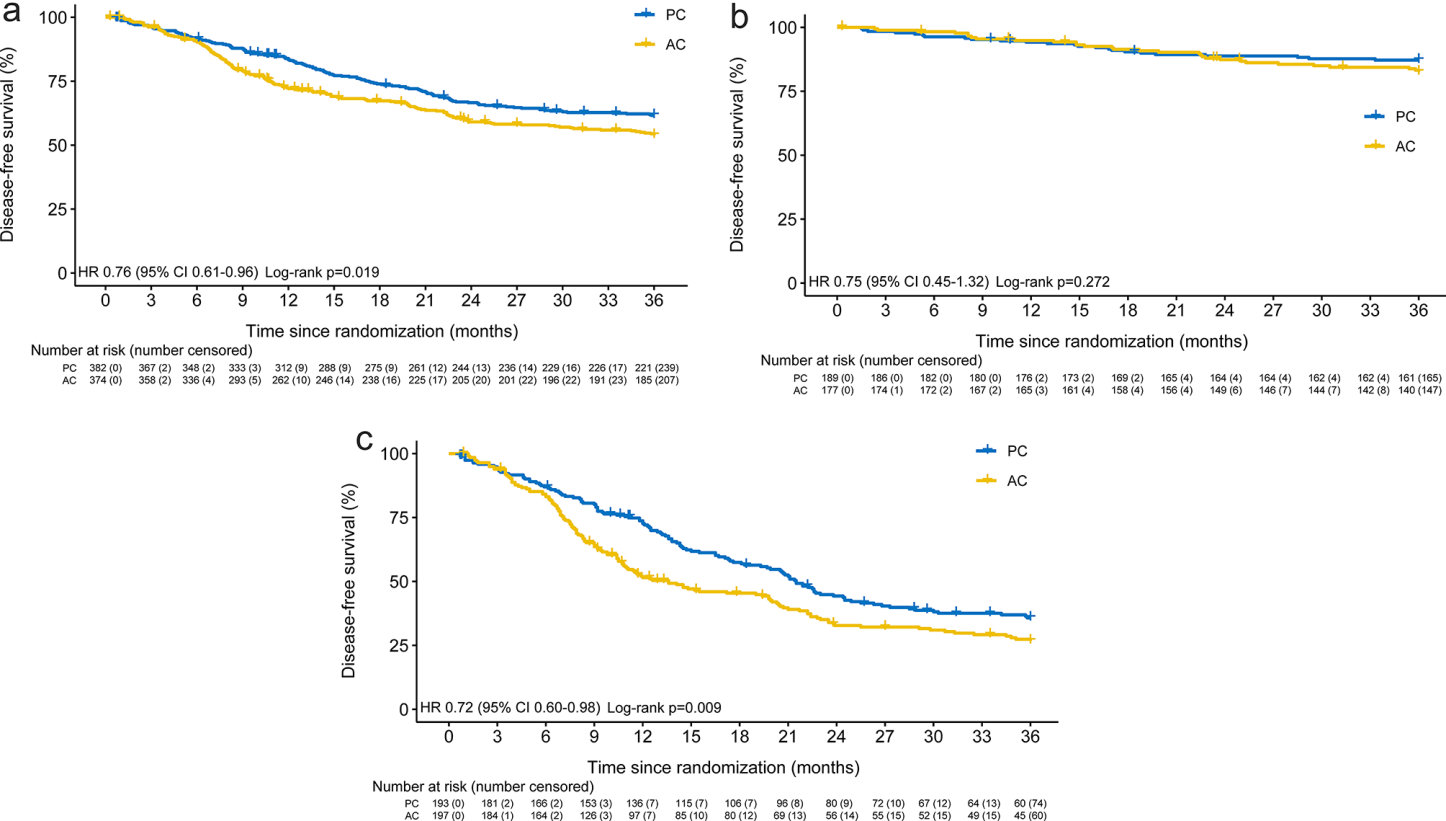
Kaplan-Meier estimates of disease-free survival for mITT patients (**A**), stage II patients (**B**), and stage III patients (**C**). HR, hazard ratio; PC, perioperative chemotherapy; AC, adjuvant chemotherapy

In the PC arm, 157 patients (41.1%) completed eight cycles of perioperative chemotherapy, while 68 (19.2%) in the AC group completed eight cycles of postoperative chemotherapy, which was significantly lower than that of the PC group (*p* < 0.001) (Additional file 2: Table S3). Preoperative chemotherapy resulted in pathological complete response (pCR) in 23.6% of patients in the PC arm. Additionally, post-hoc re-evaluation by the third party yielded a pCR rate of 22.3%.

No significant difference was found in terms of surgical time, blood loss, gastrectomy, number of dissected lymph nodes, and lymphadenectomy (Additional file 2: Table S4). The R0 resection rate of the PC group was 94.9%, which was higher than that of 83.7% in the AC group. The stratified analysis revealed higher R0 resection rates in the PC arm compared to the AC arm for stage IIIC patients or patients with tumors located in the esophagogastric junction (Additional file 2: Fig. S4).

Postoperative complications occurred in 68 patients (18.1%) in the PC arm and 73 (19.5%) in the AC arm. No significant difference in postoperative hospital stays or the rate of complication was found between the two arms (Additional file 2: Table S5, Table S6). Adverse events (AE) are listed in Table 1. The most common hematological and non-hematological AE were thrombocytopenia and fatigue, respectively. Neutropenia was the most frequent AE in all observed grade 3/4 AE. Two patients from PC group and one patient from AC group died from thrombotic event, cardiovascular event and abdominal infection, respectively.

**Table 1:** Adverse events

	PC arm				AC arm (*N* = 354)		P value (PC-post vs. AC)	
	Preoperative (*N* = 382)		Postoperative (*N* = 364)					
	All	Grade 3/4	All	Grade 3/4	All	Grade 3/4	All	Grade 3/4
Serious adverse events	8(2.1%)	3(0.8%)	12(3.3%)	6(1.6%)	18(5.1%)	11(3.1%)	1.000	1.000
**Hematological**								
Anemia	251(65.7%)	30(7.9%)	191(52.5%)	25(6.9%)	201(56.8%)	23(6.5%)	0.246	0.842
Leukopenia	242(63.4%)	16(4.2%)	184(50.5%)	19(5.2%)	190(53.7%)	14(4.0%)	0.402	0.418
Neutropenia	209(54.7%)	75(19.6%)	173(47.5%)	54(14.8%)	162(45.8%)	67(18.9%)	0.636	0.143
Thrombocytopenia	292(76.4%)	40(10.5%)	250(68.7%)	32(8.8%)	243(68.6%)	28(7.9%)	0.991	0.670
**Non-hematological**								
Anorexia	267(69.9%)	18(4.7%)	219(60.2%)	16(4.4%)	231(65.3%)	9(2.5%)	0.159	0.176
Diarrhea	180(47.1%)	12(3.1%)	156(42.9%)	9(2.5%)	130(36.7%)	11(3.1%)	0.093	0.605
Fatigue	288(75.4%)	20(5.2%)	247(67.9%)	12(3.3%)	245(69.2%)	14(4.0%)	0.697	0.637
Mucositis	108(28.3%)	2(0.5%)	89(24.5%)	1(0.3%)	105(29.7%)	3(0.8%)	0.116	0.303
Nausea	261(68.3%)	8(2.1%)	201(55.2%)	5(1.4%)	191(54.0%)	11(3.1%)	0.734	0.116
Neuropathy	187(49.0%)	14(3.7%)	157(43.1%)	15(4.1%)	144(40.7%)	9(2.5%)	0.505	0.239
Vomitting	121(31.7%)	6(1.6%)	94(25.8%)	8(2.2%)	104(29.4%)	8(2.3%)	0.287	0.955

Chemotherapy population (patients who received at least one cycle of chemotherapy). Data are n (%). PC, perioperative chemotherapy; AC, adjuvant chemotherapy; PC-post, adverse events observed in postoperative chemotherapy in the PC group

The results of our study have suggested a tendency towards higher three-year disease-free survival rate with perioperative SOX for patients with resectable stage II/III gastric cancer compared to the adjuvant SOX. The results of the subgroup analysis provide compelling evidence supporting the recommendation in the Chinese guidelines for administering neoadjuvant chemotherapy in stage III patients [11]. The limitations of this study include potential deviations in stage or response evaluation, the absence of using Lauren’s classification and microsatellite instability status, and the uneven number of enrolled cases across different centers. Despite these, we believed that this study might provide a theoretical basis for applying perioperative SOX as a standard cure in Chinese advanced gastric cancer patients.

### Electronic supplementary material

Below is the link to the electronic supplementary material.

Supplementary Material 1

Supplementary Material 2

Supplementary Material 3

## Data Availability

The datasets used and/or analyzed during the current study are available from the corresponding author on reasonable request.

## Abbreviations

SOX: S-1 plus oxaliplatin
PC: Perioperative chemotherapy
AC: Adjuvant chemotherapy
DFS: Disease-free survival
mITT: Modified intention-to-treat
HR: Hazard ratio
pCR: Pathological complete response

## References

[1] Li GZ, Doherty GM, Wang J (2022). Surgical Management of Gastric Cancer: a review. JAMA Surg.

[2] Cunningham D, Allum WH, Stenning SP, Thompson JN, Van de Velde CJ, Nicolson M (2006). Perioperative chemotherapy versus surgery alone for resectable gastroesophageal cancer. N Engl J Med.

[3] Ychou M, Boige V, Pignon JP, Conroy T, Bouché O, Lebreton G (2011). Perioperative chemotherapy compared with surgery alone for resectable gastroesophageal adenocarcinoma: an FNCLCC and FFCD multicenter phase III trial. J Clin Oncol.

[4] Al-Batran SE, Homann N, Pauligk C, Goetze TO, Meiler J, Kasper S (2019). Perioperative chemotherapy with fluorouracil plus leucovorin, oxaliplatin, and docetaxel versus fluorouracil or capecitabine plus cisplatin and epirubicin for locally advanced, resectable gastric or gastro-oesophageal junction adenocarcinoma (FLOT4): a randomised, phase 2/3 trial. Lancet.

[5] Sasako M, Sakuramoto S, Katai H, Kinoshita T, Furukawa H, Yamaguchi T (2011). Five-year outcomes of a randomized phase III trial comparing adjuvant chemotherapy with S-1 versus surgery alone in stage II or III gastric cancer. J Clin Oncol.

[6] Noh SH, Park SR, Yang HK, Chung HC, Chung IJ, Kim SW (2014). Adjuvant capecitabine plus oxaliplatin for gastric cancer after D2 gastrectomy (CLASSIC): 5-year follow-up of an open-label, randomised phase 3 trial. Lancet Oncol.

[7] Li T, Chen L (2011). [Efficacy and safety of SOX regimen as neoadjuvant chemotherapy for advanced gastric cancer]. Zhonghua Wei Chang Wai Ke Za Zhi.

[8] Zhang X, Liang H, Li Z, Xue Y, Wang Y, Zhou Z (2021). Perioperative or postoperative adjuvant oxaliplatin with S-1 versus adjuvant oxaliplatin with capecitabine in patients with locally advanced gastric or gastro-oesophageal junction adenocarcinoma undergoing D2 gastrectomy (RESOLVE): an open-label, superiority and non-inferiority, phase 3 randomised controlled trial. Lancet Oncol.

[9] Iwatsuki M, Orita H, Kobayashi K, Hidaka S, Arigami T, Kusumoto T (2022). Phase II study of S-1 and oxaliplatin as neoadjuvant chemotherapy for locally advanced adenocarcinoma of the gastric or esophagogastric junction: KSCC1601. Gastric Cancer.

[10] Honma Y, Yamada Y, Terazawa T, Takashima A, Iwasa S, Kato K (2016). Feasibility of neoadjuvant S-1 and oxaliplatin followed by surgery for resectable advanced gastric adenocarcinoma. Surg Today.

[11] Wang FH, Zhang XT, Tang L, Wu Q, Cai MY, Li YF (2024). The Chinese Society of Clinical Oncology (CSCO): clinical guidelines for the diagnosis and treatment of gastric cancer, 2023. Cancer Commun (Lond).

