# Supplementary material for: Perioperative versus adjuvant S-1 plus oxaliplatin chemotherapy for stage II/III resectable gastric cancer (RESONANCE): a randomized, open-label, phase 3 trial
Source: J Hematol Oncol. 2024 Apr 8;17:17. doi: 10.1186/s13045-024-01536-7 (PMC11003079; doi:10.1186/s13045-024-01536-7)
Supplement: Supplementary file 1 — Supplementary Material 1 [file 13045_2024_1536_MOESM1_ESM.docx]

**Additional file 1: Study Methods**

**Study Design**

The RESONANCE trial is a randomized, multicenter, controlled trial conducted at 19 medical centers throughout China. The study was carried out in compliance with the Declaration of Helsinki and was approved by the Ethics Committee of the Chinese PLA General Hospital in Beijing (approved on February 28th, 2012).

**Participants**

Patients with histologically confirmed gastric carcinomas were first staged by computer tomography (CT), endoscopic ultrasonography (EUS), or laparoscopic exploration with peritoneal lavage (optional). Then, stage II/III (AJCC seventh edition) resectable GC, including gastroesophageal junction tumor type II or III were enrolled. Further enrollment criteria included: aged 20–75 years, ECOG performance status of 0–2, gastric cancer-associated life expectancy > six months and life expectancy associated with other severe diseases > five years, granulocyte count ≥ 1.5×10^9^/L, platelet count ≥ 100×10^9^/L, hemoglobin ≥ 90g/L, alanine aminotransferase and aspartate aminotransferase < 1.5×ULN (upper limit of normal), total bilirubin ≤ 1.0×ULN, creatinine < 1.5×ULN, PT-INR/PTT < 1.7×ULN. Patients were excluded if they had other severe diseases or laboratory parameters exceeding acceptable limits, were allergic to any of the drugs included in the chemotherapy regimens, had received any chemotherapy or any of the drugs involved in this trial for four weeks before enrollment, had received cytotoxic chemotherapy, targeted therapy, immunotherapy, or radiotherapy for gastric cancer, had a history of other malignant diseases within the previous five years, were pregnant or breast-feeding women, either had severe heart diseases, including coronary heart disease, New York Heart Association grade II or worse congestive heart failure or cardiac arrhythmia or took medications for any of these diseases above, had a history of myocardial infarction within the previous 12 months, had upper gastrointestinal obstruction or absorption anomaly, had a history of peripheral nerve disease, had a history of organ transplant, had uncontrolled infections or other diseases. All patients received both oral and written information about the study and were randomized after providing written informed consent. The sex data were collected from China resident identity cards.

**Randomization and masking**

Simple randomization was used. Enrolled patients were randomized centrally using a computer-generated randomization number table at the Department of Statistics Teaching and Research of Chinese PLA General Hospital, who didn’t take part in the following enrollment. To guarantee that the sequence was concealed until the treatment is assigned, the patient identification number and treatment allocation were provided by a central telephone-based randomization system. After the patient’s eligibility for enrollment had been assessed, a unique randomization number was sent to the participating center through the central randomization system, and the patient was assigned to the appropriate group. Treatment allocation was not masked.

**Procedures**

Perioperative Chemotherapy (PC) and Adjuvant Chemotherapy (AC)

The enrolled patients were randomly assigned to the PC arm or the AC arm of the trial. In the PC arm, patients received two to four three-week cycles of preoperative SOX chemotherapy, consisting of intravenous oxaliplatin (130 mg/m^2^) over two hours administered on day one and oral S-1 on days 1–14, at doses of 40–60 mg twice daily depending on body surface area (BSA), with patients of BSA <1.25 m^2^ receiving 40 mg; those with BSA ≥1.25 m^2^ but ≤1.5 m^2^ receiving 50 mg; and those with BSA >1.5 m^2^ receiving 60 mg. Tumor response was evaluated after two, three, and four cycles of SOX according to the Response Evaluation Criteria in Solid Tumors (RECIST 1.1) [1]. If no progression was observed, preoperative chemotherapy was continued, and they received a maximum of four cycles of chemotherapy before surgery. Surgical resection was usually performed three to four weeks after the last cycle of chemotherapy. After surgical resection, patients received four to six (a total of eight cycles perioperatively) postoperative SOX chemotherapy. In the AC arm, patients received upfront surgery followed by eight cycles of adjuvant SOX chemotherapy. The evaluation was performed every two cycles postoperatively. The number of cycles was determined by physicians based on tumor response, safety evaluation, and patients’ consent.

Toxicities were measured according to NCI Common Toxicity Criteria for Adverse Events (NCI-CTCAE, version 4.0), and monitored by an independent oncologist. Dose reductions of S-1 by 10mg bid and oxaliplatin to 100mg/m^2^ or 85mg/m^2^ were introduced in patients who had either hematological toxicities grade three and above or non-hematological toxicities grade two and higher. The dose of oxaliplatin was reduced by 25% for accumulative neurotoxicity grade two. Chemotherapy was discontinued when dose modifications did not reduce grade three or four non-neurotoxicity, and oxaliplatin was discontinued in patients with neurotoxicity of grade three or above, with the latter patients continued on S-1 only if possible.

Surgical Procedures

All 19 medical centers are tertiary hospitals with more than 300 gastrectomies performed each year. Each participating surgical team has performed over 100 laparoscopies or open gastrectomies with D2 lymphadenectomies. The extent of gastric resection and lymphadenectomy were performed as per the treatment guidelines. More than 15 lymph nodes should be harvested in each D2 gastrectomy. Digestive reconstruction was conducted at the surgeon’s discretion. Postoperative morbidity and mortality were recorded.

**Outcomes**

The primary endpoint is three-year disease-free survival (DFS) rate (the period from randomization to any recurrence, new cancer, metastases, death, or evident progression for patients who did not receive surgery). Secondary endpoints include five-year overall survival rate (the period from the time of randomization to any death), R0 resection rate, safety, and pathological complete response rate of PC group. R0 resection was defined as complete tumor resection without any microscopic residual disease. The adverse events and postoperative complications were recorded and evaluated in the safety analysis. The five-year overall survival was not reported in this article due to the insufficient follow-up time and will be reported after further follow-up.

**Statistical analysis**

Based on the data from China [2-5], it was estimated that the three-year DFS rate of patients with stage III who received radical surgery and adjuvant chemotherapy is 40%. The three-year DFS rate in the perioperative chemotherapy group with the SOX regimen was assumed to be 52%. With the use of the log-rank test, a two-sided alpha of 1%, and a statistical power of 85%, assuming two years of recruitment and an additional three years of follow-up. With a 10% rate of patient loss to follow-up, the sample size was set at 386 per arm [6].

The modified intention-to-treatment (mITT) analysis was performed on patients who received any study treatment (any preoperative chemotherapy for the PC arm and gastrectomy for the AC arm). The patients excluded from the mITT population did not receive any planned treatment after randomization. Therefore, based on the opinion from the Department of Statistics Teaching and Research of Chinese PLA General Hospital and the consensus among the participating medical centers, the mITT analysis was performed. The per-protocol (PP) analysis was performed on patients who received surgery, any preoperative and postoperative chemotherapy in the PC group or received surgery, and any postoperative chemotherapy in the AC group. Perioperative outcomes were analyzed in the population who received gastrectomy. Safety analysis was performed on the population who received at least one cycle of chemotherapy. Survival curves are estimated by the Kaplan-Meier technique and DFS was compared using a two-sided log-rank test. Prespecified subgroup analysis was conducted according to N-stage, TNM-stage, tumor type, and age, gender, T-stage, signet cells post-hoc. The Cox proportional hazard model was used for calculating HR with 95%CI according to the variables mentioned above. Chi-square test was used in the comparison of categorical data. Student t test or Mann-Whitney U test was performed in the comparison of continuous variables. The third party was invited to perform an additional post-hoc re-evaluation of the response. The data analysis will be performed using SPSS Statistics (IBM Corporation) and R version 4.3.0 (The R Foundation for Statistical Computing).

This study has been registered with ClinicalTrials.gov, number NCT01583361.

**References**

1. Eisenhauer EA, Therasse P, Bogaerts J, Schwartz LH, Sargent D, Ford R, et al. New response evaluation criteria in solid tumours: revised RECIST guideline (version 1.1). Eur J Cancer. 2009; 45(2): 228-47.

2. Xu Y, Sun Z, Wang ZN, Xu HM. [Clinicopathological characteristics and prognostic factors in patients with stage III gastric cancer]. Zhonghua Wei Chang Wai Ke Za Zhi. 2012; 15(2): 125-8.

3. Zhan YQ, Li W, Sun XW, Chen YB, Xu L, Chen G, et al. [Long-term results of surgical treatment of stomach cancer: clinical experience of forty years from Sun Yat-sen University Cancer Center]. Zhonghua Wai Ke Za Zhi. 2005; 43(17): 1109-13.

4. Wang W, Li YF, Sun XW, Chen YB, Li W, Xu DZ, et al. Prognosis of 980 patients with gastric cancer after surgical resection. Chin J Cancer. 2010; 29(11): 923-30.

5. Zhang XF, Huang CM, Lu HS, Wu XY, Wang C, Guang GX, et al. Surgical treatment and prognosis of gastric cancer in 2,613 patients. World J Gastroenterol. 2004; 10(23): 3405-8.

6. Lachin JM, Foulkes MA. Evaluation of sample size and power for analyses of survival with allowance for nonuniform patient entry, losses to follow-up, noncompliance, and stratification. Biometrics. 1986; 42(3): 507-19.
