# Supplementary material for: Perioperative versus adjuvant S-1 plus oxaliplatin chemotherapy for stage II/III resectable gastric cancer (RESONANCE): a randomized, open-label, phase 3 trial
Source: J Hematol Oncol. 2024 Apr 8;17:17. doi: 10.1186/s13045-024-01536-7 (PMC11003079; doi:10.1186/s13045-024-01536-7)
Supplement: Supplementary file 2 — Supplementary Material 2 [file 13045_2024_1536_MOESM2_ESM.docx]

**Additional file 2: Supplementary Figures and Tables**

Supplementary figures

Fig. S1 Trial profile


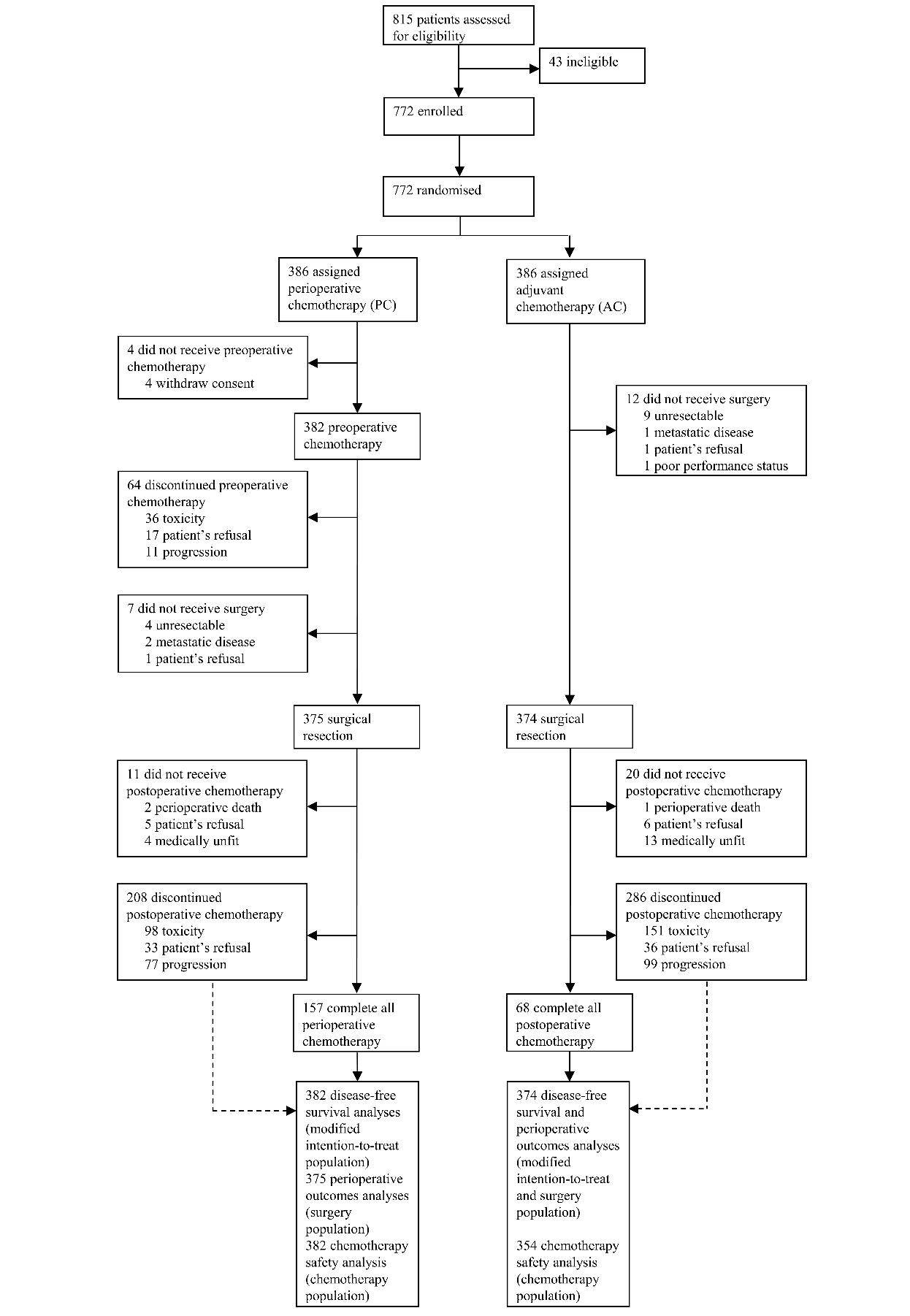


Fig. S2 Subgroup analysis: disease-free survival for mITT population


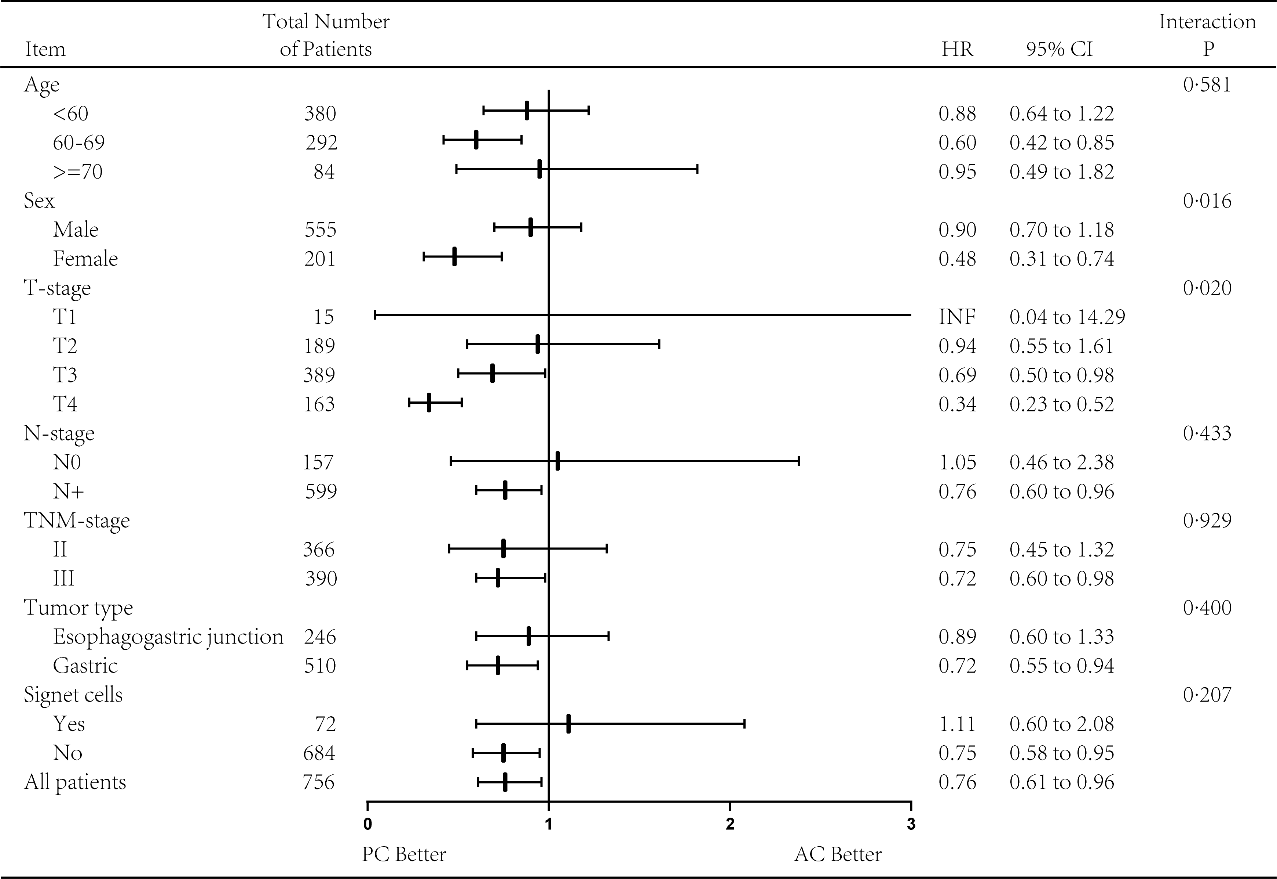


HR, hazard ratio; CI, confidence interval; PC, perioperative chemotherapy; AC, adjuvant chemotherapy.

Fig. S3 Kaplan-Meier estimates of disease-free survival for PP patients


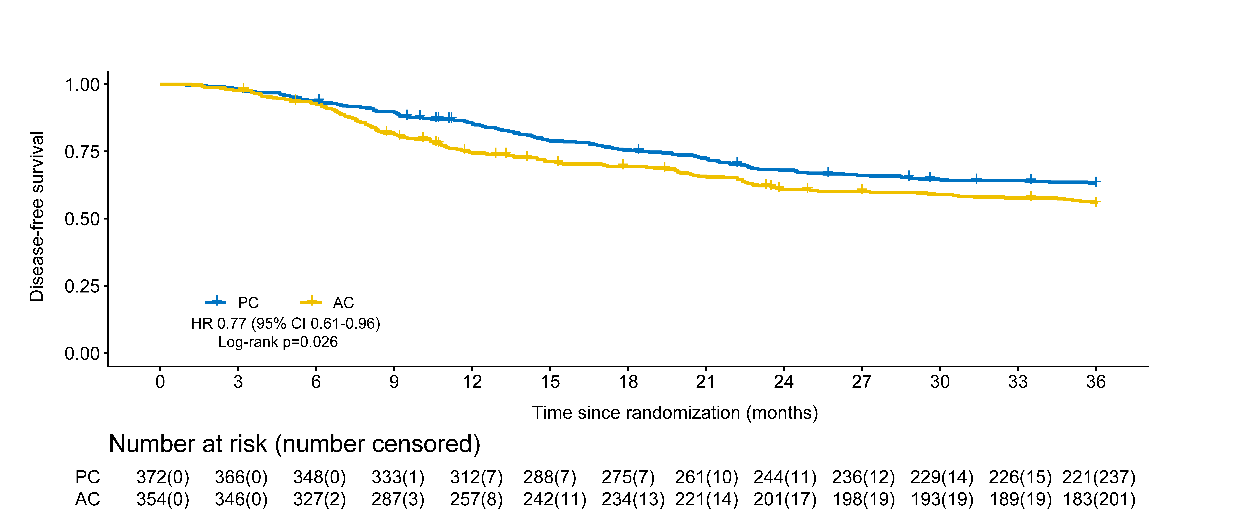


HR, hazard ratio.

Fig. S4 R0 resection rate stratified by TNM-stage and tumor type


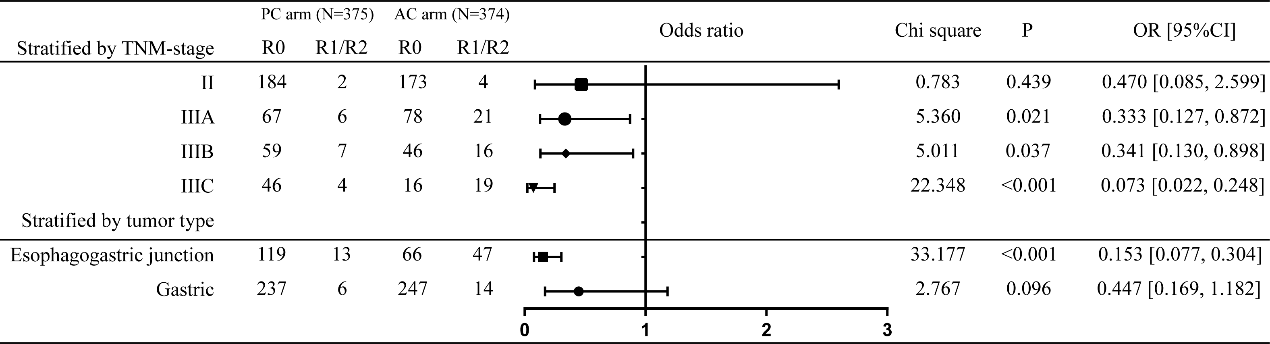


OR, odds ratio. Surgery population (patients who received gastrectomy).

Supplementary Tables

Table S1 Participant medical center

| No. | Sites |
| --- | --- |
| 1 | Chinese PLA General Hospital |
| 2 | Peking University Cancer Hospital |
| 3 | Liaoning Cancer Hospital and Institute |
| 4 | Jiangsu Province Hospital |
| 5 | Harbin Medical University Cancer Hospital |
| 6 | Peking University People’s Hospital |
| 7 | Beijing Friendship Hospital, Capital Medical University |
| 8 | Zhongshan Hospital, Fudan University |
| 9 | Tianjin Medical University Cancer Hospital |
| 10 | The First Hospital of China Medical University |
| 11 | Sir Run Run Shaw Hospital, School of Medicine, Zhejiang University |
| 12 | The Affiliated Hospital of Qingdao University |
| 13 | The First Affiliated Hospital, Sun Yat-sen University |
| 14 | Xuanwu Hospital, Capital Medical University |
| 15 | Peking University First Hospital |
| 16 | The First Affiliated Hospital of Dalian Medical University |
| 17 | Fudan University Shanghai Cancer Center |
| 18 | General Hospital of Eastern Theater Command of Chinese PLA |
| 19 | The Second Affiliated Hospital of Wenzhou Medical University |

Table S2 Baseline characteristics of modified intention-to-treat population

|  |  | PC arm  (N=382) | AC arm  (N=374) |
| --- | --- | --- | --- |
| Age |  | 60 (52-65) | 59 (51-65) |
|  | <60 | 186 (49%) | 194 (52%) |
|  | 60-69 | 151 (40%) | 141 (38%) |
|  | >=70 | 45 (11%) | 39 (10%) |
| Sex |  |  |  |
|  | Male | 276 (72%) | 279 (75%) |
|  | Female | 106 (28%) | 95 (25%) |
| ECOG PS |  |  |  |
|  | 0 | 239 (62%) | 225 (60%) |
|  | 1 | 129 (34%) | 141 (38%) |
|  | 2 | 14 (4%) | 8 (2%) |
| T-stage |  |  |  |
|  | T1 | 9 (2%) | 6 (2%) |
|  | T2 | 94 (25%) | 95 (25%) |
|  | T3 | 183 (48%) | 206 (55%) |
|  | T4 | 96 (25%) | 67 (18%) |
| N-stage |  |  |  |
|  | N0 | 87 (23%) | 70 (19%) |
|  | N+ | 295 (77%) | 304 (81%) |
| TNM-stage |  |  |  |
|  | II | 189 (49%) | 177 (47%) |
|  | III | 193 (51%) | 197 (53%) |
| Tumor type |  |  |  |
|  | Esophagogastric junction | 133 (35%) | 113 (30%) |
|  | gastric | 249 (65%) | 261 (70%) |
| Signet cells |  |  |  |
|  | Yes | 29 (8%) | 43 (12%) |
|  | No | 353 (92%) | 331 (88%) |
| Number of cycles of perioperative chemotherapy | | 7 (7-8) | 6 (4-8) |

Data are n (%) or median (IQR). PC, perioperative chemotherapy; AC, adjuvant chemotherapy.

Table S3 Chemotherapy results

|  | | PC arm (N=382) | AC arm (N=354) | P value |
| --- | --- | --- | --- | --- |
| Receive preoperative chemotherapy | | 382 (100.0%) | - |  |
|  | Complete 1 cycle | 7 (1.8%) | - |  |
|  | Complete 2 cycles | 7 (1.8%) | - |  |
|  | Complete 3 cycles | 50 (13.1%) | - |  |
|  | Complete 4 cycles | 318 (83.2%) | - |  |
| Receive postoperative chemotherapy | | 307 (80.4%) | 354 (100.0%) |  |
|  | Complete 1 cycle | 19 (5.0%) | 5 (1.4%) |  |
|  | Complete 2 cycles | 26 (6.8%) | 14 (4.0%) |  |
|  | Complete 3 cycles | 162 (42.4%) | 19 (5.4%) |  |
|  | Complete 4 cycles | 131 (34.3%) | 61 (17.2%) |  |
|  | Complete 5 cycles | 24 (6.3%) | 10 (2.8%) |  |
|  | Complete 6 cycles | 2 (0.5%) | 170 (48.0%) |  |
|  | Complete 7 cycles | - | 7 (2.0%) |  |
|  | Complete 8 cycles | - | 68 (19.2%) |  |
| Complete all perioperative chemotherapy | | 157 (41.1%) | 68 (19.2%) | <0.001 |
| Number of cycles of perioperative chemotherapy | | 7 (7-8) | 6 (4-6) | <0.001 |

Data are n (%) or median (IQR). Chemotherapy population (patients who received at least one cycle of chemotherapy).

Table S4 Perioperative outcomes

|  | | PC arm (N=375) | AC arm (N=374) | P value |
| --- | --- | --- | --- | --- |
| Surgical time(min) | | 252.6±36.8 | 247.3±39.1 | 0.062 |
| Blood loss(ml) | | 100 (80-200) | 100 (80-200) | 0.736 |
| Surgical procedures | |  |  | 0.228 |
|  | Open Surgery | 176(46.9%) | 192(51.3%) |  |
|  | Laparoscopic Surgery | 199(53.1%) | 182(48.7%) |  |
| Extent of gastrectomy | |  |  | 0.512 |
|  | Total Gastrectomy | 126(33.6%) | 131(35.0%) |  |
|  | Distal Gastrectomy | 147(39.2%) | 155(41.4%) |  |
|  | Proximal Gastrectomy | 102(27.2%) | 88(23.5%) |  |
| Number of dissected lymph nodes | | 31.6±14.1 | 32.4±13.6 | 0.380 |
| Lymphadenectomy | |  |  | 0.708 |
|  | D2 | 296(78.9%) | 291(77.8%) |  |
|  | Non-D2 | 79(21.1%) | 83(22.2%) |  |
| pTNM-stage | |  |  |  |
|  | T0N0 | 90(24.0%) | 0(0.0%) |  |
|  | I | 55(14.7%) | 23(6.1%) |  |
|  | II | 108(28.8%) | 143(38.2%) |  |
|  | III | 122(32.5%) | 208(55.6%) |  |
| Resection | |  |  | <0.0001 |
|  | R0 | 356(94.9%) | 313(83.7%) |  |
|  | R1/R2 | 19(5.1%) | 61(16.3%) |  |

Data are n(%) or mean±SD or median (IQR). Surgery population (patients who received gastrectomy).

Table S5 Postoperative complications

|  | PC arm (N=375) | AC arm (N=374) | P value |
| --- | --- | --- | --- |
| Postoperative hospital stay | 10(8-13) | 10(8-13) | 0·667 |
| Overall complications | 68(18·1%) | 73(19·5%) | 0·628 |
| Surgical complications | 52(13·9%) | 50(13·4%) | 0·843 |
| Anastomotic leakage | 8(2·1%) | 7(1·8%) | 0·798 |
| Biliary fistula | 2(0·5%) | 0(0·0%) | 0·157 |
| Chylous fistula | 5(1·3%) | 3(0·8%) | 0·479 |
| Gastrointestinal dysfunction | 16(4·3%) | 16(4·3%) | 0·994 |
| Intestinal obstruction | 0(0·0%) | 3(0·8%) | 0·082 |
| Postoperative hemorrhage | 5(1·3%) | 2(0·5%) | 0·259 |
| Wound infection | 19(5·1%) | 21(5·6%) | 0·739 |
| Non-surgical complications | 17(4·5%) | 26(7·0%) | 0·155 |
| Incision dehiscence | 0(0·0%) | 3(0·8%) | 0·082 |
| PICC infection | 3(0·8%) | 0(0·0%) | 0·083 |
| Pleural effusion | 0(0·0%) | 3(0·8%) | 0·082 |
| Pneumonia | 5(1·3%) | 5(1·3%) | 0·997 |
| Renal injury | 3(0·8%) | 4(1·1%) | 0·702 |
| Thrombus | 3(0·8%) | 4(1·1%) | 0·702 |
| Others | 5(1·3%) | 10(2·7%) | 0·190 |
| Death within 30 days after surgery | 2(0·5%) | 1(0·2%) | 0·564 |

Data are n(%) or median (IQR). PC, perioperative chemotherapy; AC, adjuvant chemotherapy. Surgery population (patients who received gastrectomy).

Table S6 Postoperative complications grade according to the Clavien-Dindo Grade

|  | PC arm (N=375) | | | | | | AC arm (N=374) | | | | | |
| --- | --- | --- | --- | --- | --- | --- | --- | --- | --- | --- | --- | --- |
|  | I | II | III | IV | V | Total | I | II | III | IV | V | Total |
| Surgical complications |  |  |  |  |  |  |  |  |  |  |  |  |
| Anastomotic leakage | 0 | 2 | 5 | 1 | 0 | 8 | 0 | 1 | 4 | 1 | 1 | 7 |
| Biliary fistula | 0 | 1 | 1 | 0 | 0 | 2 | 0 | 0 | 0 | 0 | 0 | 0 |
| Chylous fistula | 0 | 2 | 3 | 0 | 0 | 5 | 0 | 2 | 1 | 0 | 0 | 3 |
| Gastrointestinal dysfunction | 5 | 4 | 7 | 0 | 0 | 16 | 6 | 4 | 6 | 0 | 0 | 16 |
| Intestinal obstruction | 0 | 0 | 0 | 0 | 0 | 0 | 1 | 1 | 1 | 0 | 0 | 3 |
| Postoperative hemorrhage | 0 | 1 | 3 | 1 | 0 | 5 | 0 | 0 | 1 | 1 | 0 | 2 |
| Wound infection | 9 | 10 | 0 | 0 | 0 | 19 | 8 | 13 | 0 | 0 | 0 | 21 |
| Non-surgical complications |  |  |  |  |  |  |  |  |  |  |  |  |
| Incision dehiscence | 0 | 0 | 0 | 0 | 0 | 0 | 0 | 0 | 3 | 0 | 0 | 3 |
| PICC infection | 0 | 3 | 0 | 0 | 0 | 3 | 0 | 0 | 0 | 0 | 0 | 0 |
| Pleural effusion | 0 | 0 | 0 | 0 | 0 | 0 | 0 | 1 | 2 | 0 | 0 | 3 |
| Pneumonia | 0 | 5 | 0 | 0 | 0 | 5 | 0 | 4 | 0 | 1 | 0 | 5 |
| Renal injury | 0 | 2 | 0 | 1 | 0 | 3 | 0 | 3 | 0 | 1 | 0 | 4 |
| Thrombus | 0 | 2 | 0 | 0 | 1 | 3 | 0 | 3 | 1 | 0 | 0 | 4 |
| Others | 3 | 1 | 0 | 0 | 1 | 5 | 4 | 5 | 1 | 0 | 0 | 10 |

Surgery population (patients who received gastrectomy).
