# Supplementary material for: Perioperative versus adjuvant S-1 plus oxaliplatin chemotherapy for stage II/III resectable gastric cancer (RESONANCE): a randomized, open-label, phase 3 trial
Source: J Hematol Oncol. 2024 Apr 8;17:17. doi: 10.1186/s13045-024-01536-7 (PMC11003079; doi:10.1186/s13045-024-01536-7)
Supplement: Supplementary file 3 — Supplementary Material 3 [file 13045_2024_1536_MOESM3_ESM.docx]

**Additional file 3: Discussion**

The purpose of the multicenter, randomized, controlled RESONANCE study is to compare the efficacy of perioperative chemotherapy and adjuvant chemotherapy using SOX regimen in patients with locally advanced gastric cancer. Results showed that the perioperative chemotherapy using SOX was well tolerated in Chinese patients and the adverse events are acceptable. Compared with adjuvant chemotherapy, perioperative chemotherapy tended to increase DFS. The R0 resection rate was significantly higher in stage IIIC. Therefore, it was found that perioperative chemotherapy using the SOX regimen might benefit patients with locally advanced gastric cancer from a clinical perspective.

In the present study, the pCR rate of the PC group was 23.6%, which was higher than that in the FLOT4 study [1]. It may suggest that the SOX regimen seemed to be a good choice in this regard, although the relationship between pCR rate and patients’ survival has not been confirmed. However, firstly, compared with the FLOT4 study, the PC arm included more patients in a relatively early stage, which seemed to prompt us that it was one of the reasons for the high pCR rate. There may also be deviations in the stage evaluation because a small number of researchers did not conduct blind evaluation, and especially use laparoscopic exploration as an option, which may even lead to the inclusion of some stage I patients. The percentage of patients with pCR assessed by the third party was lower than that by investigators (22.3% vs 23.6%). Secondly, patients with adenocarcinoma of the esophagogastric junction (AEG) were included in the study. The fact that some patients had little response to the SOX regimen could be caused by the pathology type. Lauren’s classification and microsatellite instability status were not used in the study and may be used for further analysis.

The FLOT4 study showed that the R0 resection rates in the ECF/ECX group and FLOT group were 78% and 85%, respectively [1]. In the FNCLCC/FFCD 9703 study, 87% of the patients who underwent neoadjuvant chemotherapy with the CF regimen achieved R0 resection [2]. The JCOG0501 study is a randomized phase 3 trial from Japan that compared gastrectomy plus adjuvant chemotherapy with preoperative chemotherapy followed by gastrectomy and adjuvant chemotherapy. Short-term results showed that 80.6% of patients receiving preoperative chemotherapy with S-1/CDDP achieved R0 resection [3]. In the present study, the R0 resection rate in the PC arm was 94.9%, which was significantly higher than that in the AC arm and higher than that in the studies mentioned above. Although in the present study, CT, EUS, and diagnostic laparoscopic exploration were used to evaluate the stage, the clinical staging was still required to be more precise because a lack of precise clinical staging could result in a higher R0 resection rate.

According to the CSCO (Chinese Society of Clinical Oncology) guidelines, patients in stage cT3-4aN+M0/stage cIII are recommended neoadjuvant chemotherapy [4]. In our study, the results showed that DFS of stage III and R0 resection rates of stage IIIC in the PC group were higher than those in the AC group, which may indicate that neoadjuvant chemotherapy using SOX regimen could bring more benefits to Chinese gastric cancer patients with stage III disease.

In this study, most patients in the PC arm completed at least two cycles of preoperative chemotherapy. The ECF/ECX and FLOT regimen were performed in the FLOT4 study, in which the preoperative chemotherapy complete rates were 92% and 93% [1]. In the FNCLCC/FFCD 9703 study, patients underwent two or three cycles of preoperative chemotherapy, with a complete rate of 87% (at least two cycles of chemotherapy) [2]. The preoperative chemotherapy completion rate in our study was in line with the results from other studies, showing that the SOX regimen can be well tolerated. Patients often cannot accept all perioperative chemotherapy due to intolerance, which will affect the efficacy of perioperative chemotherapy. Just as in the AC group, the majority of patients did not complete the full 8 cycles of postoperative chemotherapy (Additional file 2: Table S3). Neoadjuvant chemotherapy facilitates the chemotherapy process and increases the completion rate of chemotherapy, thereby improving the survival of patients. However, even in the PC arm, only 41.1% of patients completed all perioperative chemotherapy in the present study, which was relatively low.

In a phase 3 study called CRITICS from the Netherlands, the ECC/EOC regimen (epirubicin, cisplatin or oxaliplatin, and capecitabine) was used in neoadjuvant chemotherapy for gastric cancer patients over 70 years old and under 70 years old. Adjuvant chemotherapy or chemoradiotherapy was followed by surgery. The results showed that 30% and 28% of patients had general postoperative complications, and 25% and 22% had surgery-related complications in the older group and younger group, respectively [5]. In the present study, the proportions of postoperative complications in the PC arm and AC arm were 18.1% and 19.5%, respectively, a result basically in line with that of the CRITICS study. The difference between proportions may be explained by the fact that our study did not perform adjuvant chemoradiotherapy but only chemotherapy. The results indicated the safety of the SOX regimen in the occurrence of postoperative complications.

The study has some limitations. Firstly, Lauren’s classification and microsatellite instability status were not used in the study. Secondly, there may also be deviations in the stage evaluation because a small number of researchers did not conduct blind evaluation and made laparoscopic exploration an option. During the evaluation process, there may have been instances where patients in a relatively early stage were included, and changes in non-target lesions and symptoms were considered for response assessment. Thirdly, the uneven number of patients in each medical center may lead to bias. The results obtained from subgroup analysis or stratified analysis still need to be validated through large sample studies. Moreover, in the sample size calculation, a two-sided alpha of 1% was set, and the p-value from the two-sided log-rank test for the comparison of DFS rate between the two groups was 0.019. From a statistical perspective, the difference in DFS did not reach significance. Therefore, in this study, we reported the p-value, described the trend of increased DFS rate from a clinical standpoint, and explained the potential clinical significance. However, further research is still needed.

The results of our study suggested a tendency towards a higher three-year disease-free survival rate with perioperative SOX in patients with resectable stage II/III gastric cancer compared to adjuvant SOX. Considering the current application of S-1 and oxaliplatin in Asian countries, as well as the feasibility of perioperative chemotherapy, which has been confirmed by large-scale clinical trials, we think that this study might provide a theoretical basis for the clinical application of SOX perioperative chemotherapy.

**References**

1. Al-Batran SE, Homann N, Pauligk C, Goetze TO, Meiler J, Kasper S, et al. Perioperative chemotherapy with fluorouracil plus leucovorin, oxaliplatin, and docetaxel versus fluorouracil or capecitabine plus cisplatin and epirubicin for locally advanced, resectable gastric or gastro-oesophageal junction adenocarcinoma (FLOT4): a randomised, phase 2/3 trial. Lancet. 2019; 393(10184): 1948-57.

2. Ychou M, Boige V, Pignon JP, Conroy T, Bouché O, Lebreton G, et al. Perioperative chemotherapy compared with surgery alone for resectable gastroesophageal adenocarcinoma: an FNCLCC and FFCD multicenter phase III trial. J Clin Oncol. 2011; 29(13): 1715-21.

3. Terashima M, Iwasaki Y, Mizusawa J, Katayama H, Nakamura K, Katai H, et al. Randomized phase III trial of gastrectomy with or without neoadjuvant S-1 plus cisplatin for type 4 or large type 3 gastric cancer, the short-term safety and surgical results: Japan Clinical Oncology Group Study (JCOG0501). Gastric Cancer. 2019; 22(5): 1044-52.

4. Wang FH, Zhang XT, Tang L, Wu Q, Cai MY, Li YF, et al. The Chinese Society of Clinical Oncology (CSCO): Clinical guidelines for the diagnosis and treatment of gastric cancer, 2023. Cancer Commun (Lond). 2024; 44(1): 127-72.

5. Slagter AE, Tudela B, van Amelsfoort RM, Sikorska K, van Sandick JW, van de Velde CJH, et al. Older versus younger adults with gastric cancer receiving perioperative treatment: Results from the CRITICS trial. Eur J Cancer. 2020; 130: 146-154.
